# Patient-Specific Quality Assurance in Pencil Beam Scanning by 2-Dimensional Array

**DOI:** 10.14338/IJPT-23-00016.1

**Published:** 2023-11-08

**Authors:** Nuttida Rawiwan, Nichakan Chatchumnan, Mananchaya Vimolnoch, Sakda Kingkaew, Sornjarod Oonsiri

**Affiliations:** 1 Medical Physics Program, Department of Radiology, Faculty of Medicine, Chulalongkorn University, Bangkok, Thailand; 2 Division of Radiation Oncology, Department of Radiology, King Chulalongkorn Memorial Hospital, Bangkok, Thailand

**Keywords:** two-dimensional ionization chamber array, pencil beam scanning proton therapy, patient-specific quality assurance

## Abstract

**Purpose:**

This study aimed to determine the characteristics of 2D ionization chamber array and the confidence limits of the gamma passing rate in pencil beam scanning proton therapy.

**Materials and Methods:**

The Varian ProBeam Compact spot-scanning system and the PTW OCTAVIUS 1500XDR array were used as a proton therapy system and detector, respectively. Our methods consisted of 2 parts: (1) the characteristics of the detector were tested and (2) patient-specific quality assurance was performed and evaluated by gamma analysis using dose-difference and distance-to-agreement criteria of 3% and 2 mm, respectively, with 123 treatment plans in head and neck, breast, chest, abdomen, and pelvic regions.

**Results:**

The PTW OCTAVIUS 1500XDR array had good reproducibility, uniformity, linearity, repetition rate, and monitor unit per spot within 0.1%, with accuracy, energy dependence, and measurement depth within 0.5%. The overall uncertainty of the PTW OCTAVIUS 1500XDR array was 2.49%. For field size and range shifter, using gamma analysis, the passing rate was 100%. The overall results of patient-specific quality assurance with the gamma evaluation were 98.9% ± 1.6% in 123 plans and confidence limit was 95.7%.

**Conclusion:**

The PTW OTAVIUS 1500XDR offered effective performance in pencil beam scanning proton therapy.

## Introduction

Proton therapy provides highly conformal dose distribution to spare normal tissues and reduce radiation side effects and secondary cancers. Generally, the beam is accelerated from 50 to 250 MeV for treatment purposes and can be delivered with 2 techniques: passive scattering and pencil beam scanning [1–3].

Currently, the most common method of delivery is pencil beam scanning, but some pencil beam scanning characteristics, particularly its finite range, can create difficulties in relation to verification. In radiation therapy, patient-specific quality assurance (QA) is important to verify the dose for delivery to avoid errors. Verification is achieved by comparing the planned dose and the measurement dose [2, 4–6].

In proton therapy, the 2-dimensional (2D) ionization chamber array is used for patient-specific QA [5, 7]. The 2D ionization chamber array is suited for routine verification of patient-specific dose distributions of the proton therapy beam [8]. Outcomes of patient-specific QA for spot-scanning proton therapy have shown that the 3%/3 mm criteria with 90% gamma passing rate is a reasonable action level for 2D comparisons of dose planes in spot-scanning proton therapy [9].

While many studies have investigated IBA MatriXX as the 2D detector for patient-specific QA in pencil beam scanning proton therapy, few have explored the PTW OTAVIUS 1500XDR. The purpose of this study was to determine the characteristics of the PTW OTAVIUS 1500XDR and the confidence limit of the gamma passing rate when using pencil beam scanning proton therapy [10].

## Materials and Methods

### Varian ProBeam Compact Spot-Scanning System

The Varian ProBeam Compact spot-scanning system (Varian Medical System, Palo Alto, California) uses the pencil beam technique [11–13].

### Detectors

#### PTW OCTAVIUS Detector 1500XDR

PTW OCTAVIUS 1500XDR (PTW-Freiburg, Freiburg, Germany) is a 2D detector array that is used for dosimetry measurements [7, 14].

#### PTW Farmer Ionization Chamber 30013

PTW30013 with PTW UNIDOS webline (PTW-Freiburg) is a standard ionization chamber in this study [15, 16].

### Treatment Planning

All treatment plans were developed with the Eclipse treatment planning system, version 16.1 (Varian Medical System).

### Dose Verification

The PTW OTAVIUS 1500XDR was verified for dose against PTW30013 with PTW UNIDOS webline in Virtual Water Phantom (Standard Imaging Inc., Middleton, USA) (slab water phantom).

### Characteristics of the Detector

Before measuring the dose distributions, the characteristics of the PTW OTAVIUS 1500XDR were tested, which was undertaken with fixed dose of 200 cGy and 160-MeV proton beams to 2-cm depth in Virtual Water Phantom with a 10 × 10-cm^2^ field.

#### Reproducibility of detector response

The detector was measured repeatedly 10 times.

#### Uniformity of detector array

The detector was measured repeatedly 3 times with a 28 × 28-cm field size.

#### Dose linearity

The linearity was measured for various dose settings ranging from 20 to 2000 cGy.

#### Accuracy of the detector array

The accuracy was measured for various dose settings ranging from 20 to 2000 cGy, and the same dose was compared against PTW30013.

#### Repetition rate

The detector was measured with different dose rates (50 000, 100 000, 750 000, 1 500 000, 3 000 000 Monitor unit per minute).

#### MU per spot

The detector was measured for various MU per spot settings ranging from 1 to 10 MU/spot.

#### Energy dependence

The detector was measured by using various energies ranging from 70 to 220 MeV (70, 100, 130, 160, 190, and 220 MeV).

#### Measurement depth

The detector was measured at 2, 3, 5, 8, 10, 15, and 20 cm with 220-MeV proton beams.

#### Field size

The detector was measured by using various field sizes ranging from 2 × 2 cm^2^ to 25 × 25 cm^2^.

#### Range shifter

The detector was measured with range shifter thicknesses of 2, 3, and 5 cm.

### Patient-Specific Quality Assurance

**Figure 1.**
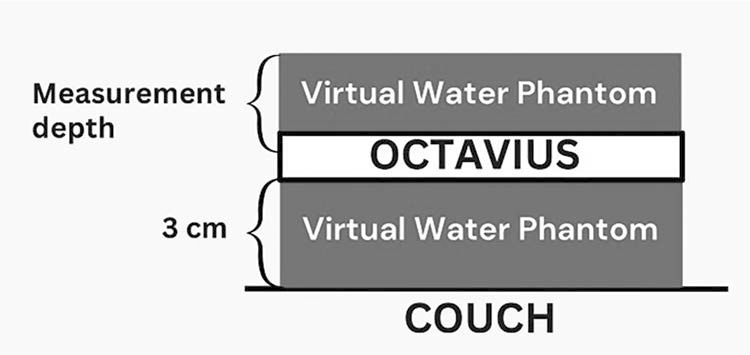
The patient-specific QA setup for each plan. Abbreviation: QA, quality assurance.

The verification plans were created with the Eclipse treatment planning system, version 16.1, in the same proton fluence as actual plans for each field. The measurement device was placed in Virtual Water Phantom in the isocenter plane with gantry at 0°. The measurement depth was selected from the same depth as the target depth as illustrated in the **Figure 1**.

The dose of each verification plan was measured and compared with the calculated dose, using the gamma index with the gamma criteria of 3%/2 mm, and 10% dose threshold using the VeriSoft (PTW-Freibrug, Freiburg, Germany) program.

The determination of the confidence limit of gamma passing rate for patient-specific QA in pencil beam scanning proton therapy was based on the formula from American Association of Physicists in Medicine Task Group 119 [17]: Confidence Limit = Mean − 1.96σ.

## Results

### Characteristics of the Detector

#### Reproducibility of detector response

The variation coefficient of reproducibility was 0.1% when compared with the specification, which was 0.5% [14], indicating excellent reproducibility.

#### Uniformity of detector array

The variation coefficient of uniformity was 0.7%, indicating uniformity.

#### Dose linearity

The linearity covers the dose range from 20 to 2000 cGy. The response of the detector was linear and the variation coefficient of linearity was 0.1%, which was within 0.5% of the specification of a detector [14].

#### Accuracy of the detector array

The variation coefficient of accuracy was 0.5% when comparing the measured dose with the dose from the ionization chamber. The difference was small—within 1.0%—indicating detector array accuracy.

#### Repetition rate

The variation coefficient of the repetition rate was 0.1%, which was within 0.5% of the specification of a detector [14].

#### MU per spot

The variation coefficient of MU per spot was 0.02%.

#### Energy dependence

The variation coefficient of energy was 0.4%, which was within 0.5%.

#### Measurement depth

The variation coefficient of measurement depth was 0.8%.

#### Field size and range shifter

When taking gamma analysis measurements at 3%/2 mm, the passing rate was 100%, with a field size of 2 × 2 cm^2^ to 25 × 25 cm^2^ and for all range shifter thicknesses.

The combined uncertainty was defined by the square root of the quadratic form of individual uncertainties. In **Table 1**, the expanded uncertainty (k = 2) was 2.49%.

**Table 1.** The uncertainty of the PTW OCTAVIUS Detector 1500 array for pencil beam scanning proton therapy.

	Uncertainty, %
Reproducibility	0.03
Uniformity	0.04
Linearity	0.02
Accuracy	0.18
Repetition rate	0.11
MU per spot	0.02
Energy dependence	0.33
Depth	0.63
N_D,W_ calibration of the user dosimeter at the standard laboratory	1.00
Combined uncertainty	1.25
Expanded uncertainty (k = 2)	2.49

### Patient-Specific Quality Assurance

**Table 2** shows that in all treatment sites, the gamma passing rate was above 95.0%; however, for the head and neck, breast, and pelvis, the gamma passing rate was above 99.0%. Overall, the gamma passing rate was 98.9% ± 1.6%.

**Table 2.** Summary of the gamma passing rate from patient-specific quality assurance of 123 treatment plans.

Treatment site	Gamma passing rate, %
	3%/2 mm
Head and neck	99.3 ± 1.3
Breast	99.6 ± 0.7
Chest	98.9 ± 1.6
Abdomen	98.1 ± 2.1
Pelvis	99.1 ± 2.1
Total	98.9 ± 1.6

Using the concept confidence limit from AAPM TG 119 [17], the results from **Table 2** show that the confidence limit was 95.7%.

## Discussion

The characteristics of the PTW OCTAVIUS 1500XDR showed its effectiveness for use in proton dosimetry. The reproducibility, linearity, and accuracy are good, demonstrating uniformity. The detector showed repetition rate, MU per spot, energy, and measurement depth independence. For field size and range shifter, the results show good agreement, with gamma passing rates of 100%. The field size and decision to use or not use a range shifter do not affect the patient-specific QA, which agreed well with the reports from Mackin et al [9] and Stelljes et al [7].

The gamma index criteria for patient-specific QA and the confidence limits of gamma passing rates in proton therapy have not been defined, so the criteria of 3%/2 mm are used. From our statistical analysis, we found that there was no significant difference between the gamma passing rate of each treatment sites, which allowed us to calculate the confidence limit for overall plans.

We felt confident using these criteria as a reference for the planning and delivery of pencil beam scanning proton therapy, as the gamma passing rate was within the confidence limit.

## Conclusion

In this study, we observe that the PTW OTAVIUS 1500XDR performed effectively and could be used for patient-specific QA in pencil beam scanning proton therapy. The confidence limit of the gamma passing rate was 95.7%. We conclude that the gamma index combining the criteria of 3%/2 mm is a reasonable action level for patient-specific QA in pencil beam scanning proton therapy. However, we use just 1 criterion for the patient-specific QA and it has a high passing rate. In a future study the criteria may be tightened, which may in turn produce effective results for patient-specific QA in proton therapy.
